# Supplementary material for: Identification of photoperiod-induced specific miRNAs in the adrenal glands of Sunite sheep (Ovis aries)
Source: Front Vet Sci. 2022 Jul 22;9:888207. doi: 10.3389/fvets.2022.888207 (PMC9354845; doi:10.3389/fvets.2022.888207)
Supplement: Supplementary file 1 [file Data_Sheet_1.ZIP › Supplementary file/miRDeep2 file.zip!/miRDeep2 file/Know_Structure/SP42A1/oar-mir-125b.pdf]

miRBase precursor	: oar-mir-125b
Total read count	: 135331
oar-miR-125b read count	: 134931
remaining reads	: 400

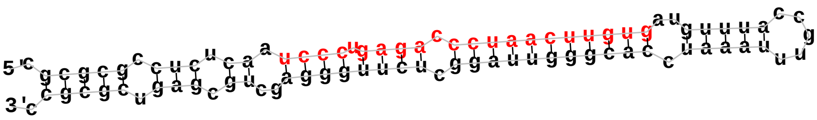

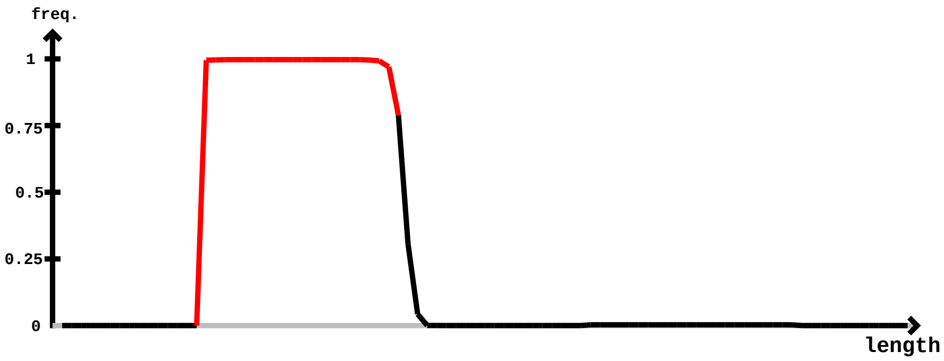

oar-miR-125b

5'- cgcgcgccucucaaucccugagacccuaacuugugauguuuaccguuuaaaauccacggguuaggcucuugggagcugcgagucgcgcc -3'	exp		
.(((((.(((.((.((((.((((.((((((((((..(((((.....))))).))))))))))).))))))))..)).))).))))).	reads	mm	sample
.............aucccugagacccuaacuugu...	1	0	seq
.............aucccugagacccuaacuugug..	1	0	seq
..............ucccugagacccuaacu..	169	0	seq
..............ucccugagacccuGacu..	1	1	seq
..............ucccugagacccuaacC..	26	1	seq
..............ucccugagacccuaacCu...	4	1	seq
..............ucccugagacccuaacuu...	432	0	seq
..............ucccuAagacccuaacuu...	2	1	seq
..............ucccugagacccuaacuug..	3023	0	seq
..............ucccugagacccuaacuugu...	24501	0	seq
..............ucccugagacccuaacuugug..	65203	0	seq
..............ucccugagacccuaacuuguga...	35501	0	seq
..............ucccugagacccuaacuugugau..	4299	0	seq
..............ucccugagacccuaacuugugaG..	1443	1	seq
..............ucccugagacccuaacuugugaGg...	13	1	seq
..............ucccugagacccuaacuugugaug...	20	0	seq
..............ucccugagacccuaacuugugaugu..	21	0	seq
..............ucccugagacccuaacuugugaGgu..	1	1	seq
..............ucccugagacccuaacuugugauguu...	1	0	seq
..............ucccugagacccuaacuugugauguuu..	1	0	seq
...............cccugagacccuaacuugu...	9	0	seq
...............cccugagacccuaacuugug..	25	0	seq
...............cccugagacccuaacuuguga...	104	0	seq
...............cccugagacccuaacuugugaG..	2	1	seq
...............cccugagacccuaacuugugau..	5	0	seq
................ccugagacccuaacuug..	3	0	seq
................ccugagacccuaacuugu...	27	0	seq
................ccugagCcccaacuugu..	1	1	seq
................ccugagaccUuaacuugu...	1	1	seq
................ccugagacccuaacuugug..	53	0	seq
................ccugagacccuaacuuguga...	31	0	seq
................ccugagacccuaacuugugaG..	2	1	seq
................ccugagacccuaacuugugau..	3	0	seq
.................cugagacccuaacuugug..	1	0	seq
.................cugagacccuaacuuguga...	1	0	seq

oar-miR-125b

```
cgcgcgccucucaaucccugagacccuaacuugugauguuuaccguuuaaauccacggguuaggcucuugggagcugcgagucgcgcc
.................cugagacccuaacuugugau...................................................   1     0     seq
.....................................................cacggguuaggcucuugggag..............   1     0     seq
.....................................................cacggguuaggcucuugggagc.............   1     0     seq
......................................................acggguuaggcucuuggg................   1     0     seq
......................................................acggguuaggcucuugggag..............   2     0     seq
......................................................acggguuaggcucuugggagc.............   29    0     seq
......................................................acggguuaggcucuugggagcu............   362   0     seq
......................................................acggguuaggcucuugggagcug...........   3     0     seq
```
